# Extended Application of Inertial Measurement Units in Biomechanics: From Activity Recognition to Force Estimation

**DOI:** 10.3390/s23094229

**Published:** 2023-04-24

**Authors:** Wenqi Liang, Fanjie Wang, Ao Fan, Wenrui Zhao, Wei Yao, Pengfei Yang

**Affiliations:** Key Laboratory for Space Bioscience and Biotechnology, School of Life Sciences, Northwestern Polytechnical University, Xi’an 710072, China

**Keywords:** inertial measurement unit, human activity recognition, joint force, ground reaction force, machine learning

## Abstract

Abnormal posture or movement is generally the indicator of musculoskeletal injuries or diseases. Mechanical forces dominate the injury and recovery processes of musculoskeletal tissue. Using kinematic data collected from wearable sensors (notably IMUs) as input, activity recognition and musculoskeletal force (typically represented by ground reaction force, joint force/torque, and muscle activity/force) estimation approaches based on machine learning models have demonstrated their superior accuracy. The purpose of the present study is to summarize recent achievements in the application of IMUs in biomechanics, with an emphasis on activity recognition and mechanical force estimation. The methodology adopted in such applications, including data pre-processing, noise suppression, classification models, force/torque estimation models, and the corresponding application effects, are reviewed. The extent of the applications of IMUs in daily activity assessment, posture assessment, disease diagnosis, rehabilitation, and exoskeleton control strategy development are illustrated and discussed. More importantly, the technical feasibility and application opportunities of musculoskeletal force prediction using IMU-based wearable devices are indicated and highlighted. With the development and application of novel adaptive networks and deep learning models, the accurate estimation of musculoskeletal forces can become a research field worthy of further attention.

## 1. Introduction

Daily human activity is characterized by a broad variety of movements which represent the capabilities of the human musculoskeletal system. An abnormal posture or movement is generally an indication of musculoskeletal injuries or diseases. Human activity recognition (HAR) is a fundamental function for identifying a specific movements or actions in an individual. It is typically based on different types of motion capture systems, wearable sensors, video, etc. Extensive state-of-the-art research has been conducted to develop various data processing and classification techniques owing to the broad application of HAR in human-to-human interactions, human-to-machine interactions, and robotics [1,2,3].

In biomechanics, the mechanical forces that originate from muscle activities are more likely to dominate development, growth, aging, and injury and recovery processes, when compared with human activities generally referred to as human kinematics. Therefore, the identification of the mechanical forces transmitted through the musculoskeletal system (represented by GRF, joint force/torque, and muscle activity/force) (i.e., human kinetics) is gaining potential and becoming clinically significant [4,5,6,7]. A growing body of research is now examining this potential and developing novel solutions to estimate the mechanical forces of the musculoskeletal system [8,9,10,11,12,13,14].

Human kinematics and kinetics have conventionally been assessed using standard systems for biomechanical data acquisition and analysis. A motion capture system embedded with a force platform and inverse dynamics analysis is the most frequently adopted approach for evaluating the kinematics and kinetics of human motion. However, the corresponding laboratory setup of the motion capture systems significantly restricts the scenarios in which it can be used. Furthermore, it involves technical difficulties in long-term monitoring during sports, rehabilitation, and outdoor activities. The inertial measurement unit (IMU) has been introduced in the past decades as one of the most representative wearable sensors to directly collect the acceleration, angular amplitude, or velocity of the human body or limb [15]. It is typically composed of an accelerometer, a gyroscope, and magnetometer sensors. IMUs are characterized by their low weight, cost effectiveness, convenience of use, and accessibility for the general population when compared with the laboratory setup. With the development of novel data processing approaches and deep learning algorithms, the capability of wearable IMUs in human kinematics and kinetics assessments has been validated in numerous studies [10,11,16,17,18,19,20].

Human activity classification based on IMU data is used to identify movement patterns. Most activity recognition models for classifying common activities performed during daily life have been established by utilizing conventional machine learning techniques, such as k-nearest neighbor (K-NN), support vector machine (SVM), random forest (RF), decision tree (DT), logistic regression, and discriminant analysis. More importantly, deep learning has gained increasing popularity in HAR tasks. In addition to the convolutional neural network (CNN) [19,20,21] and recurrent neural network (RNN) [22,23] series models, advanced approaches using attention [24,25] and transformer [26] have recently demonstrated their superior accuracy in HAR. In contrast, kinetics data can only be estimated using inverse dynamics methods based on in-lab systems for specific locomotive activities. Computational human dynamic models [27,28], neuromusculoskeletal models [29], and deep learning models [5,9,10,11,13] have been developed to calculate GRF and joint force/torque based on IMU data. Among these approaches, deep learning methods have the greatest potential to avoid subject-specific calibration and minimize sensor suite complexity.

Human kinematics and kinetics estimation using wearable sensors have been well summarized and reviewed previously. For instance, a comprehensive review on IMU-based kinetic estimation was presented, focusing on the methodologies for estimating GRF, GRM, and CoP using IMU data [30]. Another systematic review illustrated the development of GRF estimation approaches using wearable sensors, mostly based on surface electromyography sensors [31]. Furthermore, a systematic review summarized the latest progress on joint kinetic estimation using inverse dynamics and a machine learning approach with IMU data [32]. However, a comprehensive overview on the application of IMUs in biomechanics, including activity recognition, force estimation, and the corresponding applications, has not been provided.

Related research papers published in the field in the last few years were summarized and reviewed. Based on the keywords, more than a thousand related studies were collected and analyzed. Among these studies, over a hundred articles were selected and discussed in depth in four aspects: data pre-processing; machine learning and deep learning algorithms; applications and future challenges. The frequently used keywords as screening criteria employed in the present study were (“inertial measurement unit” or “accelerometer” or “gyroscope”), (“joint” or “limb” or “ankle” or “knee” or “hip”), (“kinetic” or “power” or “moment” or “torque” or “load” or “force” or “ground reaction force”), (“human activity recognition”), (“deep learning”), (“machine learning”), (“convolutional neural network” or “recurrent neural network” or “long short-term memory” or “gated recurrent unit network” or “transfer learning” or “attention” or “generative adversarial networks”). Articles that met the following criteria were excluded: studies focusing on estimating kinetic variables using sensors other than IMU as input, and articles that did not sufficiently describe the method or system.

**Figure 1:**
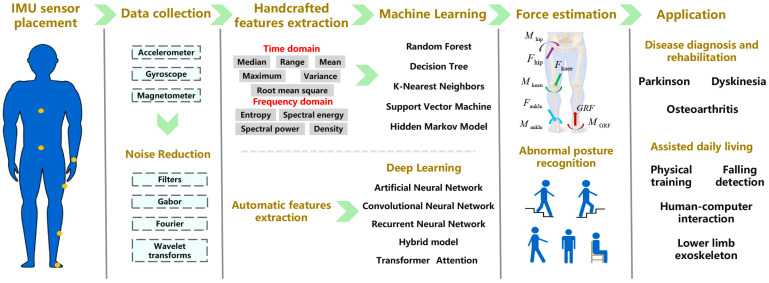
Outline of the application of IMUs in human activity recognition and musculoskeletal force estimation.

The purpose of the present study is to summarize recent progress in the application of IMUs for assessment of human kinematics and kinetics in biomechanics. The emphasis is on the methodology of activity recognition and the extended application in musculoskeletal force estimation. First, the most commonly used IMU setup, data pre-processing methods, and feature extraction for HAR and musculoskeletal force estimation were summarized. Second, classification models of HAR based on IMUs and their application in daily life and clinical diagnosis were reviewed. Third, IMU-based ground reaction force and joint force predictions during locomotive activities and their applications in rehabilitation and disease diagnosis were discussed. Finally, the upcoming challenges and opportunities for the application of IMUs in the field of biomechanics were indicated and highlighted (Figure 1).

## 2. IMU Sensor Placement and Data Acquisition and Processing

### 2.1. IMU Sensor Placement for Kinematics and Kinetics Assessments

The precision of HAR and force estimation rely substantially on the placement of the IMU sensors, particularly when using machine learning methods. This is because the estimation models learn the relationship between the input and output based on the motion data collected from the IMU of a specific position. A marginal tilt or misalignment of the sensor relative to a human limb during data collection could result in a substantial error in the derived results. The robustness of the data acquisition system reduces the risk of relative motion and misalignment between the sensors and human body.

**Figure 2:**
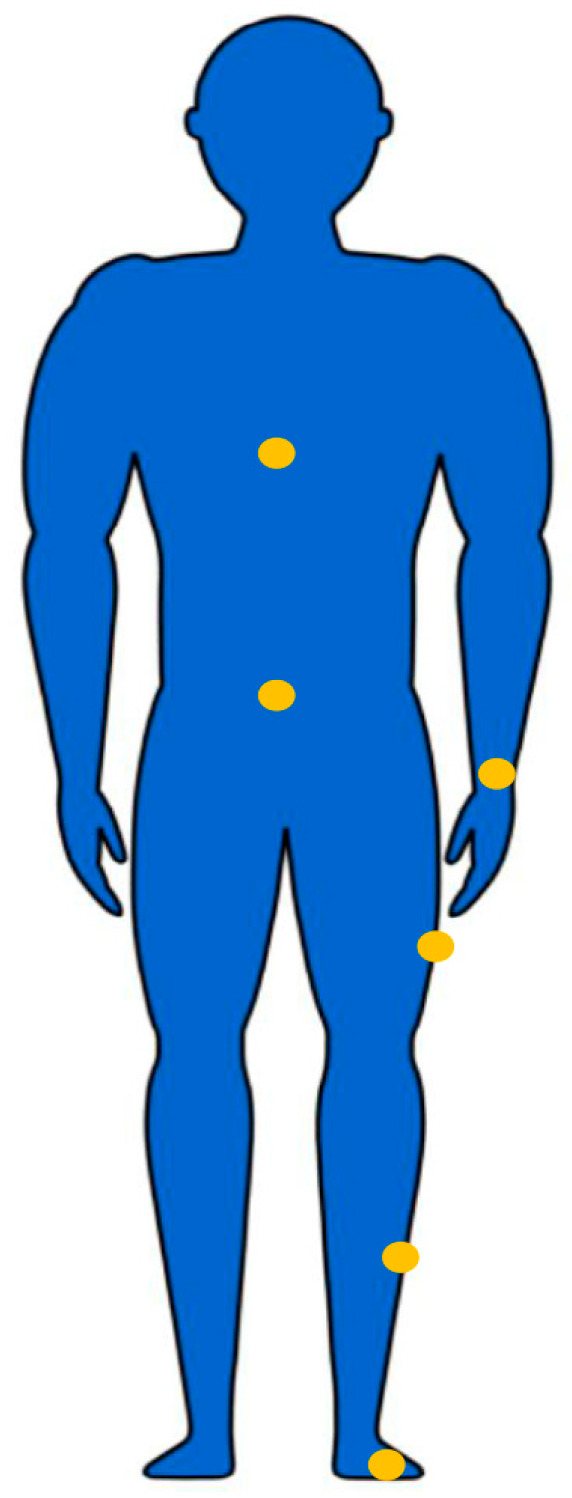
The most common locations to attach the IMUs for human activity recognition and force estimation were waist, hip, wrist, thigh, shank, and foot.

The placement of sensors for specific applications is a contentious topic. The placement setup of the sensors in different previous studies has been summarized according to the literature survey (Figure 2). Table 1 and Table 2 summarize the placement setup of IMUs in previous HAR and force estimation studies, respectively.

Body segments including the trunk, pelvis, thigh, shank, and foot are frequently selected as locations for IMUs in gait analysis [67]. It was observed that placing the accelerometer in a location with marginal acceleration variations (i.e., waist and back) yields better activity recognition accuracy than in a location with large motions. Moreover, for segments with relatively large masses (such as the trunk and pelvis), the collected vertical accelerations have been demonstrated to be highly correlated with the vertical GRF [68]. Meanwhile, the shock waves on the segments closer to the collision of each foot strike (such as the shank and foot) from impact loading are less dampened [59,69]. It is still uncertain how a precise placement and arrangement of IMUs could achieve an optimal accuracy of activity recognition and force estimation.

Numerous studies in the past decades have investigated how IMU placement could influence the accuracy of activity recognition and force estimation. Both placement and orientation errors of IMUs could result in a significant decrease in the activity recognition and force estimation [70,71,72]. An inappropriate IMU placement could also affect the magnitude and direction of the measured acceleration and angular velocity and thereby yield unreliable estimations. The misplacement of IMUs (particularly orientation placement errors) could significantly reduce GRF estimation accuracy [73]. Novel fixation solutions were developed to securely hold the IMU sensor node in a designated position and ensure the accuracy of activity recognition [74]. Moreover, an appropriate calibration procedure is an alternative method of minimizing the accuracy loss caused by the misplacement of IMUs [67,75].

### 2.2. Data Acquisition

The primary task for activity recognition or force/torque estimation is to acquire raw data or the data for a machine learning training model. Certain recent efforts have begun addressing this problem by establishing open datasets for various locomotion activities. The dataset includes IMU, EMG, and goniometer information regarding able-bodied subjects for level ground, ramps, and stairs. It can be used to train IMU-based activity recognition and force/torque estimation models [76]. For example, in the most recent open-source dataset, four IMUs were attached on the right leg to collect 3D data from 22 able-bodied adults for multiple locomotion activities (level-ground/treadmill walking, stair ascent/descent, and ramp ascent/descent) and multiple terrain conditions for each activity (walking speed, stair height, and ramp inclination). The dataset is a comprehensive source of locomotion information from the same set of subjects, which can motivate applications in activity recognition and force/torque estimation. With this dataset, the models for these applications can be subject-dependent or subject-independent. Thereby, it provides substantial flexibility for advanced research and significantly accelerates scientific achievement by fostering new analyses, good data practices, and reproducibility [77].

### 2.3. Data Pre-Processing

In general, acceleration and angular velocity data can be collected from IMUs placed on different locations of the human body. Traditional methods based on numerical calculations and machine learning approaches are the two most representative methods for IMU data processing.

Numerical methods utilized the acceleration and angular velocity collected from IMUs to calculate the orientation, velocity, and position of human limbs [78,79]. Biomechanics models were then implemented to estimate high-level parameters for HAR and musculoskeletal force estimation [80,81,82].

In order to ensure the accuracy of the calculation, bias compensation, distortion rejection, alignment, and filtering (e.g., complementary filter [83], Kalman filter [84], and zero-velocity update [85]) are normally utilized to process the raw IMU data. The most commonly used sensor fusion algorithms in the previous studies can be grouped in two main classes: Kalman filters and complementary filters. Evidence suggests that Kalman filter-based methods are computationally demanding, while complementary filter-based methods are computationally light. However, Kalman filters are more competitive for deriving more accurate results if the execution time is not considered [86].

In contrast, machine learning methods typically use raw acceleration and angular velocity measured from IMUs directly as inputs to classify activities and estimate the musculoskeletal force. Occasionally, a combination of the classic numerical calculations and machine learning methods is used to predict human kinematics and kinetics. For instance, zero velocity update was used for IMU data pre-processing, and it generated the velocity and displacement of the sacrum [9] and CoM [16]. The derived velocity and displacement data were further input into the neural network to estimate the kinematic and kinetic parameters of lower limbs.

In addition, for both activity recognition and force estimation, the noise caused by the vibration and movement of IMUs relative to the human body during data acquisition poses a challenge to the acquisition of an optimized output. Therefore, pre-processing of the raw data is a particularly critical step in the post-data mining procedure. Data pre-processing primarily includes data denoising and the calculation and extraction of eigenvalues.

The acceleration signals of human locomotive activities are primarily low frequency signals. These are non-stationary signals of a time series and normally contain random noise caused by external interference and the sensor’s vibration during the locomotive activities. This necessitates appropriate data filtering approaches. Existing solutions for filtering raw data primarily include filters and the Fourier, Gabor, and wavelet transforms. The technical details are well described elsewhere [87].

The type of eigenvalue selected is of particular importance in activity recognition and force/torque estimation. It eventually affects the accuracy of the activity recognition and force/torque estimation models. Features are abstract descriptions of the original data. Extracting features from a set of data can reduce the size of the data and result in a better comprehension of the critical information contained in the data. Conventional machine learning approaches use a set of predesigned features (also known as “shallow” features) to represent the data for activity recognition [88,89,90]. Time-domain and frequency-domain eigenvalue calculations are the main categories for feature extraction. A simple energy thresholding method was applied to the frequency analysis of input data. It was used to detect the freezing of gait in patients with Parkinson’s disease [91]. In other applications, statistical parameters, basis transform coding [89], and symbolic representation [88] were generally used as “shallow” features to describe time series data from IMUs. To simplify the machine learning model to prevent a dimension explosion, feature selection or transformation methods were the standard procedure after feature extraction. Feature selection methods can be categorized into three classes: the filter, wrapper, and hybrid methods [92]. For feature transformation, principal component analysis used an orthogonal transformation to convert raw features to compact uncorrelated new features. It was widely used to reduce the dimensions without compromising on the accuracy. For example, the nonparametric weighted feature extraction algorithm and the principal component analysis were utilized to reduce the dimensions of features and achieve a recognition accuracy of 98.23% for 10 common domestic activities [93]. A method of kernel principal component analysis and linear discriminant analysis for HAR based on smartphone IMUs was proposed to process the features further; deep belief networks were also used to train the features for activity recognition [94]. The features used in previous HAR as well as force estimation studies have been summarized in Table 3.

Although the conventional handcrafted feature learning methods are highly accessible, feature vectors extracted using such techniques are task- or application-dependent and cannot be transferred to similar tasks. Automatic feature extraction with less human effort has been achieved with the development of the machine learning approach [95]. Moreover, deep learning approaches such as CNN models are capable of automatically detecting essential features from the input data and reducing programming requirements when compared with conventional machine learning approaches [19,96,97]. Using multiple layers of abstraction, deep learning methods learn intricate features from raw sensor data and identify the most optimized pattern to improve recognition performance. Recent studies have indicated the remarkable outcome of deep learning over conventional handcrafted features for HAR [47].

**Table 3:** Selected features for HAR and musculoskeletal force estimation.

Reference	Selected Features
(De Brabandere et al., 2020) [5]	63 features generated using TSFuse (e.g., mean, median, variance, …)
(Lee, M. and Park, 2020) [9]	Position velocity and acceleration of the sacrum
(Barua et al., 2021) [12]	L2 norm and average were extracted from each accelerometer and gyroscope sensor (three axes combinedly)
(Alcantara et al., 2022) [14]	Mean, standard deviation, and range in vertical and anteroposterior acceleration data for each 12 ms window
(Derie et al., 2020) [66]	Mean, maximum, number of peaks, timing of peak values, continuous wavelet coefficients, coefficients of an autoregressive model, the time reversal symmetry statistic, Fourier coefficients
(Jiang, Napier, Hannigan, Eng, and Menon, 2020) [98]	Temporal domain features were employed, including root mean square, sum of absolute value, mean absolute deviation, variance, wavelength, slope sign changes, and simple square integral, mean wavelet with db7, difference absolute standard deviation value, average amplitude change, log detector, and the coefficients of linear fit and parabolic fit
(Jiang et al., 2019) [65]	Root mean square, sum of absolute value, mean absolute deviation, variance, wavelength, slope sign changes, simple square integral, mean wavelet with db7, difference absolute standard deviation value, average amplitude change, log detector, linear fit, and parabolic fit
(Zhu et al., 2023) [57]	PCA
(Alemayoh et al., 2021) [21]	Time-domain, frequency-domain, and wavelet transformation
(Barshan and Yüksek, 2014) [36]	The minimum and maximum values, the mean value, variance, skewness, kurtosis, autocorrelation sequence, and the peaks of the discrete Fourier transform
(Fullerton et al., 2017) [49]	Time-domain features: mean, standard deviation, root mean square, peak count, and peak amplitude Frequency-domain features: spectral energy and spectral power Heuristic features: signal magnitude area, signal vector magnitude
(Pei et al., 2013) [39]	Mean, variance, median, interquartile range, skewness, kurtosis, difference in two successive measurements, 1st dominant frequency, 2nd dominant frequency, amplitude of the 1st dominant frequency, amplitude of the 2nd dominant frequency, amplitude scale of two dominant frequencies, difference between two dominant frequencies
(Y. Liu et al., 2016) [40]	Statistical domain: mean, variance, STD, median, min, max, range, interquartile range, kurtosis, skewness Frequency domain: spectrum peak position
(Reyes-Ortiz et al., 2016) [44]	Arithmetic mean, standard deviation, median absolute deviation, largest values in array, smallest value in array, frequency signal skewness, frequency signal kurtosis, largest frequency component, average sum of the squares, signal magnitude area, signal entropy, interquartile range, 4th order burg autoregression coefficients, Pearson correlation coefficient, frequency signal weighted average, spectral energy of a frequency band [a, b], angle between signal mean and vector
(Ma et al., 2019) [46]	Spectrogram

## 3. Activity Recognition Based on IMUs

### 3.1. Classification Models

Most of the current HAR models are established using conventional machine learning approaches, including DT [33,36], K-NN [49], SVM [39,99], RF [34], Bayesian [35], and the HMM [40]. These are the most common classification algorithms with straightforward concepts and classification rules. In research using machine learning, it is important to extract handcrafted features. For this purpose, domain knowledge and signal processing theory are required. In deep learning models, the feature extraction process is performed automatically and derives sounding performance. For instance, CNN-based HAR methods could accurately extract spatial features automatically from a spectrogram converted from a time series or multivariate time series of data. A CNN model was proposed for the extraction of local features along with statistical features to obtain the global properties of the time series accelerometer data. The recognition accuracy on the public dataset Wireless Sensor Data Mining (WISDM) was 93.32% [41]. To improve the classification accuracy, a two-stage end-to-end CNN was trained using WISDM. The recognition accuracy for stair ascent and descent improved in comparison to the single-stage CNN [20]. Moreover, a double-channel CNN was trained to identify human behavior using an accelerometer and a gyroscope in a smartphone strapped to the waist. The recognition accuracy reached 97.08% using WISDM [21].

However, given that the HAR is a classification problem based on time series, the CNN-based recognition approaches were not able to capture long-term information and suffered limited performance. To overcome the shortcomings of CNN-based recognition approaches, several classical RNNs [42,50], e.g., long short-term memory (LSTM) and gate recurrent unit (GRU), were introduced for HAR. A stacked LSTM network was proposed to recognize human behavior using accelerometer and gyroscope data. The accuracy of the proposed network was 93.13% [23]. Moreover, a residual BiLSTM was proposed to resolve the gradient vanishing problem of HAR using IMU data [43].

Of note, the above-mentioned RNNs cannot effectively identify the correlations between different sensor modalities [100], leading to poor classification performance. Due to the complementary advantages of CNNs and RNNs, CNN and LSTM hybrid models were proposed and achieved notable performance [38,44]. A DeepConvLSTM model combining the LSTM model with a number of CNN layers was proposed to capture the short-term and long-term temporal correlations by learning the characterizations of the collected data. The recognition performance was a 0.69 F1 score using solely accelerometers and was improved on average by 15% when fusing accelerometers and gyroscopes; it was further elevated by 20% when fusing accelerometers, gyroscopes, and magnetic sensors [47]. Four deep learning hybrid models composed of CNN and RNN were developed to recognize complex activities. The results suggested that CNN-BiGRU achieves a recognition accuracy of 98.89%, which is better than the other three models [45].

Another significant advance in deep neural networks relevant to HAR is the use of attention and transformer [37,46,48,51]. A ConvTransformer network was first applied as the encoder of masked reconstruction-based self-supervision for HAR. This method has demonstrated its effectiveness over multiple commonly used HAR datasets [26]. A self-attention-based neural network model that foregoes recurrent architectures and utilizes different types of attention mechanisms to generate higher dimensional feature representation of IMU signals was used for classification. Results indicated that the F1 score of the model is 96% [24]. A dual-channel network consisting of a convolutional residual network, an LSTM, and an attention mechanism was proposed to process IMU data. The accuracy of the proposed network on WISDM reached 98.9% [25].

Of note, non-locomotive activities, including eating, drinking, nose blowing, reading, looking at photos, knitting, telephoning, and brushing hair, are also essential components of HAR. In an attempt to recognize activities in the dementia syndrome, an LSTM network was developed to identify nearly identical motion sequences, such as drinking, eating, writing, and nose blowing, using a smartwatch-embedded IMU. Results suggested that LSTM achieves a recognition accuracy of up to 79.81% [52]. Likewise, in combination with an LSTM network, a smartwatch was used to identify twelve daily activities, including eating with a spoon, eating with a fork, drinking from a bottle or a drinking cup, blowing the nose, cutting, writing, etc. The combination of data collected from acceleration, attitude, and gyroscope sensors achieved the best classification result with LSTM with a prediction accuracy of 85.67% [53]. Using smartwatch data, another study investigated the performance of different classifiers for four different activities including blowing the nose, cutting food, talking on the phone, and brushing hair. An accuracy of 98.33% was obtained for such activity recognition using the fast forest algorithm [54]. Moreover, logistic regression was used to classify three activities with similar motion, i.e., eating, drinking, and writing, and achieved a recognition accuracy of 98.31% [55].

It should be noted that although the deep learning algorithm has a better recognition accuracy than machine learning algorithms, it has shortcomings. First, the advantages of the deep learning algorithm rely on model training based on a large amount of data which are normally difficult to collect (particularly the high-quality and large-scale datasets). Second, most of the deep learning algorithms involve complex structures and calculation procedures. This is not conductive to certain recognition scenarios with real-time requirements and limited energy consumption. Therefore, it may be unsuitable for deployment in various devices with limited power supply.

### 3.2. Application of the IMU-Based HAR in Clinical and Daily Scenarios

Dynamic monitoring of limb movement using wireless wearable IMUs and pervasive computing technology can provide an effective and inexpensive solution for early diagnosis of musculoskeletal diseases. Data collected using wearable IMUs, in conjunction with the associated algorithms, can be mapped to make appropriate clinical assessments and provide updates on rehabilitation progression. Thus, the wearable IMU system is capable of providing valuable information to the therapist regarding the treatment solutions or training intensity during rehabilitation.

Wireless wearable devices have been used to monitor patient activities such as those with Parkinson’s disease. These can accurately quantify symptoms and motor function, support experts in clinical evaluations, and provide valuable references for rehabilitation programs. For example, data collected from two IMUs attached to both shoes were used to assess gait characteristics of Parkinson’s disease. The associated approach provided objective and quantitative results on different gait parameters that can be used as valid and reliable biofeedback in daily life. This can satisfy the requirements of physicians, patients, and caregivers [101]. A recent study used IMU to integrate technology to monitor and evaluate the gait of patients with Parkinson’s disease. An IMU integrated with a cloud computing approach has demonstrated its capability for monitoring freezing episodes and reducing variability or the risk of falling. For patients with Parkinson’s disease, a single IMU system could improve the follow-up of the recovery process after they receive treatment [102]. With the widespread application of the machine learning approach, the corresponding models and algorithms are also becoming highly effective tools to process the clinical data obtained by IMUs. A study with two IMUs mounted on the shank was used to analyze gait patterns of healthy subjects and patients with peripheral neuropathy, post-stroke, or Parkinson’s disease. Eight spatiotemporal and kinematic gait parameters were extracted and classified with an accuracy of 93.9% using an SVM algorithm. This indicates the feasibility of gait pattern recognition with a few IMUs and its significant potential in clinical applications [103]. Similarly, with a deep convolutional neural network and IMU-based sensor system, the rehabilitation process can be classified with an accuracy of up to 98% while walking at an arbitrary speed on flat ground [104]. It is therefore indicated that the IMU-based approach could be one of the objective, evidence-based solutions for clinical diagnosis and rehabilitation evaluation through the recognition of human movement patterns.

Similarly, IMU-based activity recognition has also been used in daily life. Falling is one of the leading causes of injuries among the elderly. Numerous studies have been conducted using wearable IMUs for fall detection and fall prediction. A three-axis accelerometer and HMM computing approach can detect and predict fall events with an accuracy of 100% [105]. Timely assistance in case of a fall and a reduction in casualty rates are becoming feasible with the emergence of wireless transmission-combined IMU systems and positioning technologies. For example, a low-cost multifunctional wearable IMU-based device was developed for real-time location and movement activity monitoring, particularly for fall detection and emergency notification for the elderly [106].

Posture assessment of human daily behavioral patterns generally requires precise capturing of posture during locomotive tasks. Using the information exported by IMUs, coaches can accurately evaluate the performance of athletes and develop appropriate training programs [107]. Similarly, training and match demands were quantified using the jumping count data obtained from IMUs. This aided the reduction in knee joint injuries among volleyball players [108].

In the field of human–computer interactions, IMUs were used to monitor the movement of the human body and convey the corresponding information to the machine. This can be applied in various scenarios of the industrial field [109,110]. A kinematics-based approach was developed to estimate human leg posture and velocity from IMUs during the performance of typical physiotherapy and training exercises. The proposed approach used an extended Kalman filter to estimate joint angles from accelerometer and gyroscopic data. It was capable of deriving joint angles from arbitrary 3D motion [110]. In a recent study, bidirectional long short-term memory, CNN, a wavelet neural network, and LSTM networks were trained to estimate lower limb joint angles using a single IMU attached to the pelvis. The results suggested that the LSTM networks derive a better estimation with a minimum error of 5.8° and a maximum error of 11.32° [111]. In a subsequent study, a multi-joint angle estimation approach based on an LSTM network with a single shank-attached low-sampling-frequency (23 Hz) IMU was proposed. The results showed that the estimation accuracy is comparable to the previous studies using high-frequency IMU sensors. The estimated angle coefficient of determination was greater than 0.74. The root mean square error and normalized root mean square error were less than 7° and 9.87%, respectively [112]. An IMU-based virtual reality therapy system has the potential to increase the intensity and frequency of physical activity at home for stroke patients. The virtual reality system uses three IMUs attached to the lower and upper arm and the trunk to capture patient motion data for training upper extremity functions. This may help increase the dose of rehabilitation without the costs associated with clinical visits and therapist supervision [109].

## 4. Estimation of Musculoskeletal Force Using IMUs

The application of IMUs and deep learning techniques is exhibiting increasing potential and clinical significance. Essentially, all human activities are the result of a combination of muscle forces and gravitational forces. Therefore, an estimation of the mechanical forces in the musculoskeletal system (represented by ground reaction force, joint force/torque, and muscle activity/force) would be more straightforward than activity recognition in clinical diagnosis, rehabilitation evaluation, and daily locomotive activity monitoring [113].

Several methods have been established to calculate GRF or joint forces using data collected by IMUs. Inverse dynamics and machine learning techniques are a few of the most representative methods for such purposes. Inverse dynamics is one of the classical methods for calculating the lower limb joint load based on IMU data. The inverse dynamics approach assumes that the human body is a multi-link rigid segment system connected by joints and the recursive Newton–Euler inverse dynamics formula. Thereafter, the kinematic and kinetics variables of human motion can be calculated. This procedure requires a number of assumptions, which introduces inaccuracies and uncertainty [80]. In contrast, machine learning is a potential method for solving the above-mentioned problems. The machine learning approach could accomplish self-learning and model construction based on sample data (so-called training data) and makes predictions or decisions without explicit programming. Force or torque prediction based on IMUs has become feasible and accurate with the development of machine learning and deep learning approaches and their application in the field of biomechanics. Table 4 and Table 5 summarize the accuracy of GFR and joint force estimation achieved in different studies with different IMU numbers and placements in the human body.

### 4.1. Estimation of Musculoskeletal Force Using IMUs

The GRF is one of the most representative parameters for motion analyses. As an external force in the most distal link of the human lower limb, GRF is used in inverse dynamics analyses to calculate continuous joint dynamics including the force and torque of each link of the human body. The vertical component of the GRF during running can be used to predict the occurrence risk of knee injury in long-distance runners [66]. Furthermore, the GRF during walking can also be used to assess fall risk and abnormal gait [114].

A series of data-driven methods have been developed to estimate GRFs. These include the conventional regression models and the recently developed deep learning methods. DT-based methods have been widely used owing to their substantial capability for representing the relationships in the data. DTs are inherently transparent in their decision-making process. This highly valuable feature can provide information regarding which joints are critical for estimating the GRF. Using data derived from two shin-mounted tri-axial accelerometers and a gradient-boosted regression tree model machine learning approach, the maximal vertical instantaneous loading rate was predicted with high precision (mean absolute error of 12.41 ± 7.90 BW·) during overground running [66]. An RF is also a supervised classification algorithm consisting of a large number of decision trees. A novel solution using an RF algorithm was proposed to estimate the timing and magnitude of the vertical GRF peaks during walking using the data from an IMU fixed on one of the lower limbs. The proposed method achieved a significantly high correlation coefficient (R = 0.97) averaged over all the speeds (0.4 m/s, 0.7 m/s, 1.0 m/s, 1.3 m/s, and 1.6 m/s) [98].

The most evident feature of the nonlinear auto-regressive moving average model with exogenous inputs (NARMAX) modeling methodology is that it produces transparent mathematical functions that are directly related to the task. NARMAX methods provide linear and/or nonlinear dynamic relationships and models between user-defined inputs and outputs [115]. Therefore, it is appropriate to apply NARMAX to determine the optimal location of IMUs when IMU data are used for force estimation. NARMAX was used to identify the optimal number and location of IMUs on different body segments to accurately estimate the vertical, medial–lateral (ML), and anterior–posterior (AP) GRFs. A set of 12 IMUs was used to measure the tri-axial acceleration and orientation signals at the seventh cervical vertebrae (C7), fifth lumbar vertebrae (L5), upper arms, fore arms, thighs, shanks, and fourth metatarsals. The best locations for IMUs to estimate the three components of the GRF were C7 for the single-sensor system; C7 and L5 for the two-sensor system; and C7, L5, and one of the thighs for the three-sensor system. In all the cases, the higher the number of sensors, the lower the NRMSE of the estimated GRF signals. The average decrease in these NRMSEs with an increase in the number of sensors from one to three were 2%, 3%, 3%, and 4% for the V, AP, ML, and tri-axial directions, respectively. A simple linear model was then proposed to estimate the GRF. It was observed that for the three-IMU system, the proposed model estimated the GRF with average NRMSEs of 7%, 13%, and 13% in the vertical, AP, and ML directions, respectively [59]. In another study, NARMAX and the orthogonal forward regression algorithm were used to predict the vertical GRF from IMUs attached at different locations including L5, C7, and the forehead. The results indicated that the prediction accuracy was better for the IMU at L5 than for the sensors on C7 and the forehead. The vertical GRF was reconstructed with high accuracy and an average prediction error of less than 5.0% when only one wearable sensor mounted at the waist (L5) was used [58].

The ANN is one of the earliest neural network models used to predict GRF. MLP is the most commonly used ANN. It displays high computing speed, is convenient to implement, and has small training set requirements. Two concatenated ANNs were trained using an ambulatory minimal IMU setup to estimate the kinematics and kinetics of runners. The first ANN mapped the orientation and acceleration of three IMUs on the lower legs and pelvis to lower-body joint angles. The estimated joint angles in combination with the measured vertical accelerations were input into a second ANN that estimated the vertical GRF. The results indicated that the mean RMSE of the estimation was less than 0.27 BW [56]. A system based on an IMU and an ANN was developed to estimate vertical GRF waveforms during running and metrics during jumping (peak force, flight time, peak power at landing, etc.). During running, the system relies on two IMUs located on the left and right shanks. Meanwhile, only one IMU worn on the pelvis is required for jumping. The predictions were satisfactory (RMSE of vertical GRF during running = 0.148 BW; error < 10% for most jumping metrics) [64]. The evidence indicated that the GRF could be predicted with moderate effectiveness using an ANN, based on the dynamic relationship between the center of mass (CoM), the GRF, and joint kinetics. The vertical GRF can be predicted with a correlation coefficient of 0.97 using an IMU at the CoM in combination with an ANN [16]. An IMU at the sacrum in combination with an ANN predicts the 3D GRF, CoP trajectory, and joint torque of the lower extremity with moderate accuracy. The normalized root mean square errors range from 6.7% to 15.6%, 8.2% to 20.0%, and 11.4% to 24.1%, respectively [9].

CNN models are highly effective for image classification. In addition, they can accurately and automatically extract spatial features from time series data. In the past, CNN models have been widely used for HAR based on IMU data. Recently, these have been trained to predict GRFs. Two CNN models, CaffeNet and ResNet-50, were trained using laboratory-derived stance phase GRF or moment data; they simulated IMU output during running and sidestepping maneuvers obtained from nearly half a million legacy motion trials. The proposed deep learning workbench achieved high correlations (>0.87) with the ground truth [8]. LSTM networks have been used in GRF prediction because of their advantages in extracting long-term dependence within time series. A hybrid model consisting of a bidirectional LSTM and an MLP with three fully connected layers containing 128, 384, and 320 neurons was developed to predict continuous vertical GRFs across a range of running speeds and slopes from sacral- and shoe-mounted accelerometer data. The results indicated that the hybrid model predicted with a higher accuracy than the neural networks implemented in the previous studies [14]. A transformer encoder was trained to extract temporal and spatial features from IMU data to estimate GRF. Five IMUs worn on the pelvis, left thigh, left ankle, right thigh, and right ankle were used to collect kinematics data. The results showed that the average error of the predicted values was improved by 32% when compared to the RNN architecture and by 25% when compared to the LSTM architecture, while using the transformer as a feature extractor [57].

### 4.2. Estimation of Joint Force and Moment of Lower Limb Based on IMUs

To determine joint forces and moments with IMUs, various biomechanical models, including segmented 2D/3D models, were established for kinematics and kinetics calculations [80,81,116,117,118]. However, most of the biomechanical model-based methods require the placement of IMUs onto each segment of the lower limb, making them too cumbersome for application in daily continuous monitoring. Machine learning approaches have the greatest potential to avoid subject-specific calibration and minimize sensor suite complexity.

The application of conventional machine learning algorithms in joint force/moment estimation is constrained by extensive feature extraction requirements and longer data pre-processing. Meanwhile, deep learning could simplify this process and perform better and faster (which is essential for real-time estimations). An RF learning algorithm was employed with two wireless IMUs to train and test the ankle power prediction model. The results indicated that the accuracy of the intra-subject tests was 0.98 across the five speeds of gait. Meanwhile, the accuracy of the inter-subject test was 0.92, which is marginally lower than that of the intra-subject test. Although the estimation of the ankle joint power using two IMUs and the RF algorithm was reasonably effective, it generally required a considerable amount of feature extraction (256 features) and could not perform well in peak power [65]. To overcome the shortcomings of the RF model, three state-of-the-art deep neural architectures (namely, LSTM, CNN, and their hybrid CNN-LSTM) were developed, trained, and evaluated for predicting ankle joint power. Using two IMUs attached at the foot and shank, the proposed hybrid architectures were found to be efficient and have potential, with a considerably high estimation accuracy (correlation coefficient R > 0.92 and adjusted R-squared value > 83%) and low errors (mean squared error < 0.06 and mean absolute error < 0.13) in inter-participant evaluations [12].

The recent advancement in deep learning has shifted research interest from conventional machine learning approaches toward deep learning techniques. A literature survey indicated that the ANN is the most commonly used model for predicting joint forces. More recently, joint force prediction was conducted with CNN, RNN, and a hybrid model using IMU data. In a validation study, the performance of three machine learning models commonly employed for predicting gait kinematics and kinetics (namely, MLP, LSTM, and CNN) were compared. CNNs appeared to be favorable for predicting joint angles. In contrast, the ANNs did not show an advantage over the MLP network for the prediction of joint moments. The LSTM network was more effective for real-time joint angle and joint moment prediction [13].

An ANN with two hidden layers (one with 250 neurons and another with 100 neurons) was developed to calculate the knee joint forces (KJFs) in motions of straight-line gait, gait direction change, and jumping. The ANN-predicted knee joint forces yielded correlation coefficients that ranged from 0.60 to 0.94 for vertical KJFs, 0.64 to 0.90 for anterior–posterior KJFs, and 0.25–0.60 for medial–lateral KJFs. The estimated force in the sagittal and horizontal planes of the knee joint was in good agreement with the reference during most exercises [62]. In a subsequent study, an ANN with two hidden layers of 100 and 20 neurons was developed based on the input of the right thigh and calf IMUs to calculate knee flexion moments (KFMs) and knee adduction moments (KAMs) during six locomotion tasks. For all the locomotion tasks, the RMSE for KFM was between 0.26 ± 0.09 and 1.13 ± 0.46 Nm/kg; for KAM, it was between 0.18 ± 0.06 and 0.92 ± 0.54 Nm/kg. The results indicated the feasibility of using only two IMUs for estimating KAMs and KFMs during locomotive activities [4].

**Figure 3:**
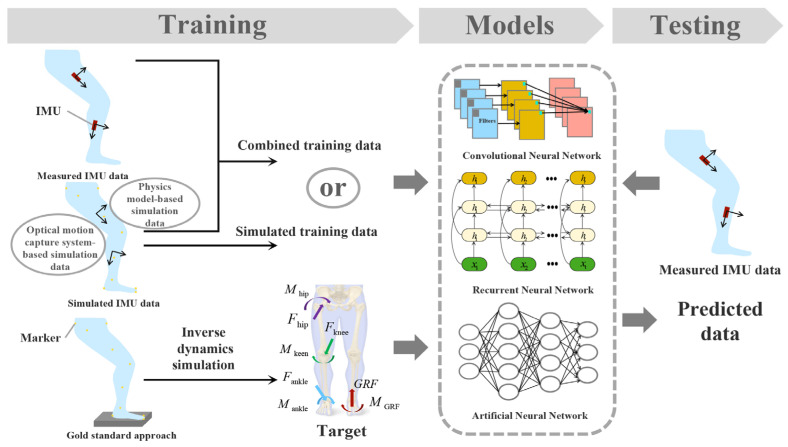
Overview of force estimation using simulated IMU data.

More complex deep learning models were normally effective for improving the estimation accuracy. However, more training data was also required. The lack of sufficient training data for deep learning models has become an issue owing to the limitations of sample size or data resources. To overcome this limitation, simulated IMU data was generated from optical motion capture data for model training (Figure 3). A 2D CNN consisting of two convolutional layers was trained to learn the mapping rules between IMU data and sagittal plane kinetics variables. In this work, physics-based model simulation data was used to reduce the exhaustive tasks of training data collection and training data-driven models that could provide low-latency feedback. After the addition of the simulated data to the training dataset, the correlation coefficients increased marginally from 0.970 to 0.971 for the knee joint moment and from 0.983 to 0.985 for the vertical GRF [10]. Alternatively, acceleration and angular data were obtained from a marker-based optical motion tracking system to simulate the IMU data of different sensor positions/orientations and to increase the size of the database. Gait analysis was undertaken with 30 participants using a conventional motion capture set-up based on an optoelectronic system and force plates in parallel with a custom IMU system consisting of five sensors. The correlation coefficients for the joint angles increased from 0.85 to 0.89 after the addition of the simulated data to the training dataset. Meanwhile, the correlation coefficient of the joint moments remained at 0.95. The enlargement of the dataset improved the prediction of the joint angles. It was therefore indicated that appropriate augmentation techniques to process the raw data could be effective for further improving machine learning applications [11]. More recently, a musculoskeletal model was used to generate the data for virtual IMUs attached to the trunk and thigh. A TCN framework was then trained to estimate the hip joint moment using realistic hip goniometer data and data collected by two virtual IMUs during walking. The average estimation RMSE values of the obtained results were 0.131 ± 0.018 Nm/kg and 0.152 ± 0.027 Nm/kg during steady-state ambulation and mode transitions, respectively. However, in practice, factors such as soft tissue deformation, changes in the subject anthropometry, and sensor noise may introduce error into the IMU data, placing greater demands on the robustness of the deep learning model [61]. By leveraging different conventional deep learning layers, i.e., 1D, 2D convolutional, GRU, and dense layers, a novel deep learning model using ensemble learning bagging was proposed. Hip, knee, and ankle joint moments and GRF were estimated using three IMUs attached to the thigh, shank, and foot during treadmill, level-ground, stair, and ramp walking. The results suggested that the average PCC for joint moments and 3-dimensional GRF estimation is 0.90 [63].

Ideally, the IMU-based deep learning approaches have significant potential to be applied in many more scenarios. Minimizing the number of IMUs while maintaining sufficient accuracy is becoming essential. To assess the feasibility of joint loading prediction with a low number of sensors and high portability, an ANN was proposed to predict joint moments and GRFs using a single, sacrum-mounted IMU with extracted features, including acceleration, velocity, displacement, and time. The predicted joint moments of the stance leg yielded normalized root mean square errors ranging from 9.24 ± 1.91% to 11.67 ± 2.03% [16]. Likewise, an ANN was established to estimate 3D lower limb kinetics during walking using sacrum-attached IMU data. The normalized root mean square errors of the predicted lower limb joint torques were in the 11.4–24.1% range [9]. Another interesting study developed a method for predicting human kinetics using an IMU-embedded mobile phone to collect data during a variety of locomotive activities. A linear regression model was trained with a dataset of nine exercises to estimate hip kinetics. It was found that the prediction errors for the left and right hips were 29% and 36%, respectively. However, the results also indicated that the use of a single IMU may still pose a technical challenge in obtaining improved performance of such prediction [5]. In summary, the potential application of IMU-based deep learning methods in the evaluation of lower limb posture and joint mechanical loads is highly anticipated.

### 4.3. Clinical Application of IMU-Based Data Analysis Approaches

Weight bearing and intense exercise cause a higher proportion of joint injuries in the population. Long-term joint overloading or transient excessive joint loading is one of the causes of joint injury [119]. Therefore, the development of a smart sensor system or hardware to reduce joint loading has important clinical implications. Over the past few decades, exoskeletons have been developed for injury protection or rehabilitation training. For example, the lower limb exoskeleton has become a strong potential solution for restoring lower limb mobility in patients [120].

As one of the most efficient solutions for collecting kinematics and kinetics data of human motion, IMUs were also used in exoskeleton development. The knee torques required to maintain segment balance and the desired assistance was estimated using two force–torque sensors and two IMUs attached to the subject shank. A certain percentage of the estimated knee torque was then input as a reference assistive torque to the embedded torque controller to drive the dual actuators of the knee’s exoskeleton [121]. A novel joint muscular torque estimation method based on inverse dynamics calculations, a sensing system with IMU, and 1D and 3D force sensors were presented to calculate the assistive torque and achieve an active power-assist function on an active power-assist lower limb exoskeleton. The proposed method successfully acquired the joint torque of the human body. The integration of sensors into the active power-assist lower limb system ensures its portability [6]. A novel motor intent decoding scheme was proposed and validated using IMUs to generate a fully customized assistive force profile. After improving the robustness of the IMU-based kinematic estimation approach, a computationally efficient dynamic model was developed using the estimated kinematics as input. Furthermore, the kinetics of subjects was calculated in real time. The results of the test on the ankle exoskeleton showed that the fully customized assistive force profile enabled by a motor intent decoding scheme can provide effective assistance [7].

Clinically, IMUs and the associated machine learning models and algorithms were also utilized for knee osteoarthritis (KOA) diagnosis and treatment. KFMs and KAMs constitute objective parameters of knee joint load in KOA. An ANN was developed to estimate the aforementioned parameters based on time series data obtained by two IMUs located on the right thigh and shank. For all the six locomotion tasks, the ANN achieved high overall concordance in KAM (r = 0.39 ± 0.32, rRSME = 29.9 ± 8.1%) and KFM (r = 0.74 ± 0.36, rRSME = 20.8 ± 5.7%). This is essential for providing valuable biofeedback systems to KOA patients [4]. A multi-layer SVM-based online segmentation model was proposed. It achieved a segmentation accuracy of 92.7%. Although this method is more accurate, a larger number of IMUs are required for joint loading estimation [122]. The model is also effective for hip OA patients and can be monitored using a mobile phone attached to their hips. An ML pipeline using only an embedded IMU was proposed for training the musculoskeletal model to estimate the loading value. The proposed linear regression-based pipeline achieves mean absolute errors of 29% and 36% for the left and right hips, respectively [5]. Rehabilitation exercise plays an important role in KOA therapy. Motion segmentation is the main difficulty in rehabilitation monitoring. An ML-based grading system that can predict the post-intervention response to exercise therapy through IMU data was established to assess the performance of muscle strength training. The overall accuracy in performance was up to 81.7% with IMUs installed on the back, thigh, and shank of the human body [123].

## 5. Discussion, Challenges, and Outlook

### 5.1. Discussion of Reviewed IMU-Based Systems

#### 5.1.1. Data Recording, Quantity and Placement of IMUs

Most of the HAR research summarized in the present study used benchmark data for model training, while only a few studies utilized the self-collected data. For HAR, the WISDM [124] and UCI datasets [125] are the most commonly used datasets established using the IMUs embedded in smartphones. Wrist, arm, ankle, and chest are the most common locations to attach IMUs for HAR. In contrast, for musculoskeletal force estimation, nearly all of the model training processes were based on self-collected IMU data and kinetics data derived from inverse dynamics calculations, as the corresponding open-source dataset has not yet been well developed.

Sampling frequency, number, and placement of sensors are some of the main factors in determining the estimation accuracy of HAR and the musculoskeletal force [126]. In general, higher sampling frequency could facilitate the derivation of more accurate models at the cost of higher energy and resource consumption. Human activities are normally low frequency signals in the range 0–20 Hz [127]. Comparatively, in previous studies, the sampling frequency of most IMUs for HAR remained at 25–100 Hz. Of note, the sampling rates of more than 50% of the IMU-based force estimation systems were in the range of 100–250 Hz [9,12,13,16,56,57,58,59,63,64,65,98], with one exception below 100 Hz [5]. Eight force estimation studies used IMUs with a sampling rate above 1000 Hz [4,10,14,60,62,66]. It is therefore conceived that a sampling frequency of 50 Hz is generally sufficient for accurate human activity detection [127], while a higher sampling frequency is required for musculoskeletal force estimation.

Likewise, musculoskeletal force estimation studies summarized in the present paper tend to use a larger number of IMUs than HAR. Most previous studies used four or five IMUs attached to the human body. Of the 26 musculoskeletal force estimation studies discussed in the present paper, 19 studies used more than one IMU to capture limb motion, particularly for the estimation of joint force, while a single IMU may still pose a technical challenge in obtaining acceptable performance for such predictions.

Furthermore, the placement of IMUs determines the eventual output of the system. Vertical acceleration of relatively large mass limbs has been demonstrated to be highly correlated with the vertical GRF [68]. Therefore, most GRF estimation studies attached IMUs to the pelvis [5,56,57], CoM [16], and the sacrum [9,14]. For the estimation of lower limb joint force/moments, IMUs attached to the shank seem to be able to achieve better estimation accuracy [63]. Of the 14 studies for joint force/moments estimation summarized in the present paper, ten studies further added the shank-attached IMU [2,4,10,11,12,13,60,62,63,65], and seven studies used a combination of the thigh- and shank-attached IMUs [4,10,11,13,60,62,63].

#### 5.1.2. Potential of IMUs for HAR and Force Estimation

The characteristics of wearability, portability, and intelligence of IMU-based systems provide their broad application possibilities for daily activity monitoring and clinical diagnosis.

IMU-based HAR technologies have been widely used in the field of sports for detecting sport conditions, monitoring the players in sports, aiding referees, and in entertainment systems to interact with computer games in fun ways [102,103,104,106,107,108,109]. Almost all major consumer electronics companies worldwide have launched their own IMU-based wearable products, such as the Apple watch, the Microsoft band, and the Huawei watch. However, despite the large number of wearable motion monitoring devices that have emerged, there are still some technical challenges and shortcomings to be overcome, including signal quality, miniaturization, and data acquisition techniques.

For musculoskeletal force estimation, only limited applications have been found in previous studies on clinical monitoring, diagnosis, and rehabilitation assessment [4,5,102,103,104,122,123]. It is worth noting that insufficient clinical data and research are limiting IMU-based systems to become commercial products and enter the market for force estimation.

With the emerging techniques of artificial intelligence and big data processing capabilities, it can be anticipated that accurate quantitative analysis of human activity and the musculoskeletal force based on IMUs can be achieved in the future.

### 5.2. Challenges and Opportunities for Implementation of the Deep Learning Method in IMU-Based Systems

The rapid development of deep learning models dramatically improved the accuracy of IMU-based HAR and musculoskeletal force estimation. However, there are still several challenges to be overcome. Collection of sufficient data and development of robust and sensitive deep learning models are the two main limitations to improving the accuracy of HAR and musculoskeletal force estimation.

#### 5.2.1. Dataset for Deep Learning Methods

Data are the cornerstone of deep learning model establishment and play a decisive role in model performance. To build generalizable models, more attention should be paid to data collection by ensuring that the participants are representative of the population of interest. However, most public datasets only present data from healthy subjects; this impairs the usability of these models in clinical scenarios. Data collection requires a considerable amount of effort, particularly for musculoskeletal force estimation, and labelling of the data is very time consuming.

To solve the problem of small sample size of the labeled training data in supervised learning, optical motion capture data have been used to generate simulated IMU data for force estimation model training [8,11,61]. However, the reliability of the model trained by simulated IMU data as compared to real IMU data is unclear because noise can be easily introduced by the skin when using moving artifacts during IMU data acquisition. Recently, generative models such as GANs are being widely used to generate time series fabricated data [128,129,130]. These studies provide inspiration for using GANs to address the problem of lack of data for improving the performance of musculoskeletal force estimation models.

#### 5.2.2. Development of Novel Deep Learning Models

The robustness and sensitivity of the system need to be analyzed and optimized further in the big data environment. Most of the HAR and musculoskeletal force estimation systems were based on data collected in experimental environments. However, real-world activities are more complex due to occlusion, interference, and noise and thus harder to model. For instance, the type of surface, clothing, and previous history of injuries or surgeries affect the activity of individuals in real life.

Novel adaptive networks and deep learning models should be more widely applied and optimized. This would ultimately provide more accurate information of human activities and force estimation. The most significant advance in deep neural networks relevant to force estimation is the use of attention, transformer, and transfer learning. In recent years, attention and transformers have been used in time series-related studies [24,26]. Strategies such as transfer learning have been applied to improve robustness and generalization of data-based models. However, these models are not yet applied in the estimation of the musculoskeletal force.

### 5.3. Outlook

To study the neural control of movement, it is generally necessary to estimate how muscles are activated across a variety of behavioral conditions. Therefore, it is necessary to comprehensively assess the muscle activity/force of the hamstring tendon, quadriceps femoris, gastrocnemius, and other key muscle groups of the lower extremities during exercise using deep learning methods. Owing to the time series characteristic of the muscle signal, RNNs are reasonably effective for identifying the timing of muscle activity/force. Compared with the traditional unsupervised characterization of complex dynamical systems, more advanced training approaches, e.g., sequential autoencoders and large-scale optimization approaches, would facilitate RNNs to achieve better performance during the muscle activity/force prediction tasks. EMG is susceptible to powerline noise, movement artifact, and cross-talk. Deep learning models are highly sensitive to even marginal amounts of artifacts. Hence, the intelligent selection of the signals extracted from EMG to mitigate the potentially hazardous effects of these noise sources and predict muscle activity accurately and comprehensively is a problem that needs to be addressed. Moreover, the design and provision of mobile applications and devices to collect, process, and predict patient data in an automated manner using the collected kinematic data and an appropriate model to classify motion would be important. The subsequent step would be to select an appropriate kinetic prediction model based on classification results for kinetic analysis. Eventually, an initial report of the musculoskeletal force of the individual subject would be generated. Given that the muscle force is the origin of locomotive activities, the use of IMUs to solve muscle forces would be a future research direction with high potential.

## 6. Conclusions

This study presents a comprehensive overview of the application of IMUs in both activity recognition and force estimation in the field of biomechanics. More specifically, the present study summarizes recent research progress in data collection, processing, human activity classification models, machine learning models, and the estimation of ground reaction forces and the joint force/moment using IMUs. The applications of IMUs and the associated data analysis methodologies for daily activity assessment, posture assessment, human–computer interactions, disease diagnosis, rehabilitation, and exoskeleton control strategy development are illustrated. Moreover, the present limitations and challenges of the research field are indicated. More importantly, the growth potential and opportunities for predicting the forces of the musculoskeletal system using IMU-based wearable devices and their future application in daily activity monitoring, clinical diagnosis, and treatment are discussed. Generally speaking, mechanical forces that originate from muscle activities and gravitational loading are more directly related to musculoskeletal injury and recovery processes. This has superior implications for clinical diagnosis and rehabilitation assessment when compared to human activity recognition. With the development and application of wearable technologies and novel deep learning models, accurate estimation of the musculoskeletal force using IMUs should gain increasing attention from research.

## Figures and Tables

**Table 1:** IMU locations adopted in the previous studies for human activity recognition.

Location	References
Thigh	(Barshan and Yüksek, 2014; Liu, S. et al., 2020; Martinez-Hernandez and Dehghani-Sanij, 2018; Rezaie and Ghassemian, 2017; Safi, Attal, Mohammed, Khalil, and Amirat, 2015) [33,34,35,36,37]
Shank	(Liu, S. et al., 2020; Martinez-Hernandez and Dehghani-Sanij, 2018) [35,37]
Phone	(Abdel-Basset et al., 2021; Alemayoh et al., 2021; Yuwen Chen, Zhong, Zhang, Sun, and Zhao, 2016; Haresamudram et al., 2020; Ignatov, 2018; Liu, Y., Zhao, Shao, and Luo, 2016; Ma, Li, Zhang, Gao, and Lu, 2019; Mekruksavanich and Jitpattanakul, 2021; Pei et al., 2013; Reyes-Ortiz, Oneto, Samà, Parra, and Anguita, 2016; Yao, S., Hu, Zhao, Zhang, and Abdelzaher, 2017; Zhao, Yang, Chevalier, Xu, and Zhang, 2018) [21,25,26,38,39,40,41,42,43,44,45,46]
Waist	(Haresamudram et al., 2020; Liu, S. et al., 2020; Mahmud et al., 2020) [24,26,37]
Foot	(Abedin, Ehsanpour, Shi, Rezatofighi, and Ranasinghe, 2021; Mahmud et al., 2020; Martinez-Hernandez and Dehghani-Sanij, 2018; Ordóñez and Roggen, 2016; Zhao et al., 2018) [24,35,43,47,48]
Arm	(Abedin et al., 2021; Fullerton, Heller, and Munoz-Organero, 2017; Guan and Ploetz, 2017; Liu, S. et al., 2020; Ma et al., 2019; Mahmud et al., 2020; Ordóñez and Roggen, 2016; Ming Zeng et al., 2018; Zhao et al., 2018) [24,37,43,46,47,48,49,50,51]
Chest	(Abedin et al., 2021; Barshan and Yüksek, 2014; Guan and Ploetz, 2017; S. Liu et al., 2020; Ma et al., 2019; Mahmud et al., 2020; Rezaie and Ghassemian, 2017; Safi et al., 2015; Ming Zeng et al., 2018) [24,33,34,36,37,46,48,50,51]
Wrist	(Abedin et al., 2021; Barshan and Yüksek, 2014; Fullerton et al., 2017; Guan and Ploetz, 2017; Hassemer et al., 2023; Liu, S. et al., 2020; Ma et al., 2019; Mahmud et al., 2020; Mekruksavanich and Jitpattanakul, 2021; Rezaie and Ghassemian, 2017; Sergio Staab, Bröning, Luderschmidt, and Martin, 2022; S. Staab, Krissel, Luderschmidt, and Martin, 2022; Staab, S., Luderschmidt, and Martin, 2021; Ming Zeng et al., 2018) [24,33,36,37,45,46,48,49,50,51,52,53,54,55]
Ankle	(Abedin et al., 2021; Fullerton et al., 2017; Guan and Ploetz, 2017; Liu, S. et al., 2020; Ma et al., 2019; Mahmud et al., 2020; Safi et al., 2015; Ming Zeng et al., 2018) [24,34,37,46,48,49,50,51]
Hip	(Fullerton et al., 2017) [49]
Spine	(Fullerton et al., 2017) [49]
Head	(Liu, S. et al., 2020) [37]
Trunk	(Liu, S. et al., 2020; Ming Zeng et al., 2018) [37,51]

**Table 2:** IMU locations adopted in the previous studies for estimating ground reaction force and joint force/moment.

Location	References
CoM	(Hyerim Lim et al., 2020) [16]
Sacrum	(Alcantara et al., 2022; M. Lee and Park, 2020) [9,14]
Pelvis	(Johnson et al., 2021; Mundt et al., 2021; Mundt et al., 2020; Wouda et al., 2018; Zhu, Xia, and Zhang, 2023) [8,11,13,56,57]
L5	(Guo et al., 2017; Shahabpoor et al., 2018) [58,59]
C7	(Shahabpoor et al., 2018) [59]
Lower back	(Dorschky et al., 2020) [10]
Thigh	(Chaaban et al., 2021; Dorschky et al., 2020; Hossain, Guo, and Choi, 2023; Johnson et al., 2021; Molinaro, Kang, Camargo, Gombolay, and Young, 2022; Mundt et al., 2021; Mundt et al., 2020; Shahabpoor et al., 2018; Stetter et al., 2020; Stetter, Ringhof, Krafft, Sell, and Stein, 2019; Zhu et al., 2023) [4,8,10,11,13,57,59,60,61,62,63]
Shank	(Barua et al., 2021; Chaaban et al., 2021; Derie et al., 2020; Dorschky et al., 2020; Hossain et al., 2023; Jiang, Gholami, Khoshnam, Eng, and Menon, 2019; Johnson et al., 2021; Mundt et al., 2021; Mundt et al., 2020; Stetter et al., 2020; Stetter et al., 2019; Tedesco et al., 2021) [4,8,10,11,12,13,60,62,63,64,65,66]
Phone	(De Brabandere et al., 2020) [5]
Foot	(Alcantara et al., 2022; Barua et al., 2021; Dorschky et al., 2020; Hossain et al., 2023; Jiang et al., 2019; Zhu et al., 2023) [10,12,14,57,63,65]
Hip	(De Brabandere et al., 2020; Molinaro et al., 2022) [5,61]

**Table 4:** Model selection and IMU sensor position for estimating ground reaction force.

Reference	Number of Sensors	Position	Frequency	Activation	Tasks	Optimal Model	Accuracy
(Guo et al., 2017) [58]	1	L5	128 Hz	Walking	vGRF	OFR	Average prediction percentage error < 5%
(Wouda et al., 2018) [56]	3	Pelvis, lower legs	240 Hz	Running	vGRF	ANN	Single subject training: ρ > 0.99, Multiple subject training: ρ > 0.9
(Shahabpoor et al., 2018) [59]	3	C7, L5, thigh	128 Hz	Walking	Tri-Axial GRF	Linear regression	NRMSE: vGRF: 7%, A-P GRF: 16%, M-L GRF: 18%
(Lim et al., 2019) [16]	1	CoM	100 Hz	Walking	A-P GRF, vGRF	ANN	The approximate errors: vGRF: 58 N, A-P GRF: 23 N
(Johnson et al., 2021) [8]	5	Pelvis, thigh, shank	Virtual IMU data	Sidestepping, running	Tri-Axial GRF	CaffeNet	Pearson correlation coefficient: 0.89
						ResNet-50	Pearson correlation coefficient: 0.87
(Jiang et al., 2020) [98]	1	Shank	100 Hz	Walking	vGRF	Random forest regressor	Intra-subject test: RMSE = 0.02 BW Inter-subject test: RMSE = 0.10 BW
(Lee and Park, 2020) [9]	1	Sacrum	148 Hz	Walking	Tri-Axial GRF	ANN	NRMSE: Tri-Axial GRF: 6.7–15.6%,
(Dorschky et al., 2020) [10]	4	Lower back, the right thigh, shank and foot	1000 Hz	Walking, Running	A-P GRF, vGRF	CNN	Pearson correlation coefficients: A-P GRF: 0.970 vGRF: 0.980
(Chaaban et al., 2021) [60]	4 acc, 4 gre	Thigh, shank	1125 Hz	Jumping	vGRF	Linear regression	RMSE: 0.22 ± 0.002 BW
	4 acc						RMSE: 0.25 ± 0.003 BW
(Tedesco et al., 2021) [64]	2	Left and right shanks	238 Hz	Running	vGRF	ANN	RMSE: 0.148 BW
(Alcantara et al., 2022) [14]	3	Two on the right shoe and one on the sacrum	2000 Hz	Uphill and downhill running	Perpendicular to running surface GRF	RNN	RMSE: 0.16 ± 0.04 BW
(Zhu et al., 2023) [57]	5	Pelvis, left thigh, left ankle, right thigh, and right ankle	100 Hz	Fast running, jogging, slow walking, brisk walking, and walking up and down stairs	vGRF	Transformer	MSE: 0.0205

OFR: Orthogonal forward regression, vGRF: Vertical ground reaction force, A-P: Anterior–posterior ground reaction force, CoM: Center of mass, L5: 5th lumbar vertebrae, C7: 7th cervical vertebrae, NRMSE: Normalized root mean square error, RMSE: Root mean square error, MSE: Mean squared error.

**Table 5:** Model selection and IMU sensor position for estimating joint force/torque.

Reference	Number of Sensors	Position	Frequency	Activation	Tasks	Optimal Model	Accuracy
(Stetter et al., 2019) [62]	2	Right thigh and shank	1500 Hz	Walking, jumping	KJF	ANN	Pearson correlation coefficients: vertical KJF: 0.60–0.94, P KJF: 0.64–0.90, M-L KJF: 0.25–0.60.
(Lim et al., 2020) [16]	1	CoM	100 Hz	Walking	Joint torques	ANN	The approximate errors: hip joint torques: 16.7 Nm, knee joint torques: 11.4 Nm, ankle joint torques: 15.3 Nm.
(Jiang et al., 2019) [65]	2	Shank, foot	100 Hz	Walking	Ankle joint power	RF	Intra-subject test: R = 0.98, Inter-subject test: R = 0.92.
(Derie et al., 2020) [66]	2	Antero-medial side of both tibias	1000 Hz	Running	Maximal vertical loading rate	XGB	Subject-dependent: mean absolute percentage error: 6.08%, Subject-independent: mean absolute percentage error: 11.09%.
(Lee and Park, 2020) [9]	1	Sacrum	148 Hz	Walking	Joint torques	ANN	NRMSE: joint torques: 11.4–24.1%
(Stetter et al., 2020) [4]	2	Right thigh and shank	1500 Hz	Walking, running, 45° cutting maneuver	KFM, KAM	ANN	KFM: R = 0.74 ± 0.36, KAM: R = 0.39 ± 0.32.
(De Brabandere et al., 2020) [5]	1	Left hip	50 Hz	Walking, walking upstairs/downstairs, sitting down and standing up, forward lunge and side lunging, standing on one leg, squatting on one leg	Hip moment	Regularized linear regression	Mean absolute error: left hip: 29%, right hip: 36%.
(Dorschky et al., 2020) [10]	4	Lower back, the right thigh, shank and foot	1000 Hz	Walking, running	Joint moments	CNN	Pearson correlation coefficients: hip moment: 0.94, knee moment:0.975, ankle moment: 0.981.
(Mundt et al., 2020) [11]	5	Pelvis, thigh, shank	Virtual IMU data	Walking	Joint moments	MLP	The mean correlation of the models: r-kinetic-measured: 0.95, r-kinetic-combined: 0.95.
(Barua et al., 2021) [12]	2	Foot, shank	100 Hz	Walking	Ankle joint power	LSTM	R > 81.25%
						CNN	R > 83.09%
						CNN-LSTM	R > 83.19%
(Chaaban et al., 2021) [60]	4 acc, 4 gre	Thigh, shank	1125 Hz	Jumping	Knee extension moment, sagittal plane knee power absorption	Linear regression	RMSE: knee extension moment: 0.028 ± 0.0002 BW·HT, sagittal plane knee power: 0.27 ± 0.003 BW·HT.
	4 acc						RMSE: knee extension moment: 0.031 ± 0.0002 BW·HT sagittal plane knee power: 0.32 ± 0.003 BW·HT
(Mundt et al., 2021) [13]	5	Pelvis, thigh, shank	100 Hz	Walking	Joint moments	MLP, LSTM, CNN	Mean model correlation coefficients: joint moment > 0.939.
(Molinaro et al., 2022) [61]	3	Trunk, thigh, and hip	Virtual IMU data	Walking	Hip moment	TCN	Average RMSE: steady-state ambulation: 0.131 ± 0.018 Nm/kg, mode transitions: 0.152 ± 0.027 Nm/kg.
(Hossain et al., 2023) [63]	3	Thigh, shank, and foot	100 Hz	Tread-mill walking, level-ground walking, ramp ascent/descent, and stair ascent/descent	Hip, knee, and ankle joint moment, 3D GRFs	Hybrid model based on 1D, 2D convolutional, GRU, and dense layers with the application of bagging techniques	PCC: 0.923 ± 0.030
	8	Trunk, pelvis, and both thighs, shanks	100 Hz	Walking	KFM, KAM, and 3D GRFs		PCC: 0.884 ± 0.029

KJF: Knee joint force, KFM: Knee flexion moment, KAM: Knee adduction moment, NRMSE: Normalized root mean square error, RMSE: Root mean square error, TCN: Temporal convolutional network.

## Data Availability

Not applicable.
